# Poly (ADP-ribose) polymerase plays an important role in intermittent hypoxia-induced cell death in rat cerebellar granule cells

**DOI:** 10.1186/1423-0127-19-29

**Published:** 2012-03-09

**Authors:** Sheng-Chun Chiu, Sung-Ying Huang, Yu-Chieh Tsai, Shee-Ping Chen, Cheng-Yoong Pang, Chih-Feng Lien, Yu-Jou Lin, Kun-Ta Yang

**Affiliations:** 1 Department of Medical Research, Buddhist Tzu Chi General Hospital, Hualien, Taiwan; 2 Department of Ophthalmology, Mackay Memorial Hospital, Hsinchu, Taiwan; 3 Department of Optometry, Jen-Teh Junior College of Medicine, Nursing and Management, Miaoli, Taiwan; 4 Physiological and Anatomical Medicine, School of Medicine, Tzu Chi University, Hualien, Taiwan; 5 Tzu Chi Stem Cells Center, Buddhist Tzu Chi General Hospital, Hualien, Taiwan; 6 Institute of Medical Sciences, School of Medicine, Tzu Chi University, Hualien, Taiwan; 7 Department of Physiology, School of Medicine, Tzu Chi University, Hualien, Taiwan; 8 Tzu Chi University, No.701, Zhongyang Rd., Sec .3, Hualien 97004, Taiwan

**Keywords:** Intermittent hypoxia, Oxidative stress, Poly (ADP-ribose) polymerase, Calpain, Cerebellar granule cell

## Abstract

**Background:**

Episodic cessation of airflow during sleep in patients with sleep apnea syndrome results in intermittent hypoxia (IH). Our aim was to investigate the effects of IH on cerebellar granule cells and to identify the mechanism of IH-induced cell death.

**Methods:**

Cerebellar granule cells were freshly prepared from neonatal Sprague-Dawley rats. IH was created by culturing the cerebellar granule cells in the incubators with oscillating O_2 _concentration at 20% and 5% every 30 min for 1-4 days. The results of this study are based on image analysis using a confocal microscope and associated software. Cellular oxidative stress increased with increase in IH. In addition, the occurrence of cell death (apoptosis and necrosis) increased as the duration of IH increased, but decreased in the presence of an iron chelator (phenanthroline) or poly (ADP-ribose) polymerase (PARP) inhibitors [3-aminobenzamide (3-AB) and DPQ]. The fluorescence of caspase-3 remained the same regardless of the duration of IH, and Western blots did not detect activation of caspase-3. However, IH increased the ratio of apoptosis-inducing factor (AIF) translocation to the nucleus, while PARP inhibitors (3-AB) reduced this ratio.

**Results:**

According to our findings, IH increased oxidative stress and subsequently leading to cell death. This effect was at least partially mediated by PARP activation, resulting in ATP depletion, calpain activation leading to AIF translocation to the nucleus.

**Conclusions:**

We suggest that IH induces cell death in rat primary cerebellar granule cells by stimulating oxidative stress PARP-mediated calpain and AIF activation.

## Background

Sleep apnea is a major public health problem in Western and Asian countries [1]. Obstructive sleep apnea [2] is the most prevalent type of sleep apnea. Patients with OSA are at increased risk of cardiovascular diseases and neuro-cognitive deficits [3,4]. Magnetic resonance imaging studies in OSA patients have revealed significant reductions in gray matter of several brain regions, including the cortex, hippocampus, and cerebellum [5].

Episodic cessation of airflow during sleep in patients with OSA results in intermittent hypoxia (IH) [1], which cycles through periods of hypoxia and reoxygenation. Reoxygenation increases the risk of oxidative stress and cell injury [6]. Oxidative stress results from the presence of excessive reactive oxygen species (ROS), including superoxide (O_2_^-^·), hydrogen peroxide (H_2_O_2_), and the hydroxyl radical (OH ·). Excessive ROS is associated with aging, cardiovascular disease, and neuronal diseases.

Hypoxia or hypoxia-reoxygenation injury results in neuronal cell death, including apoptosis and necrosis. Oxidative stress results in mitochondrial dysfunction and the release of cytochrome *c *and apoptosis-inducing factor (AIF), which are associated with apoptosis through caspase-dependent and caspase-independent pathways, respectively [7]. In case of mild DNA damage, the cell activates poly(ADP-ribose) polymerase (PARP) to facilitate DNA repair [8,9]. Severe DNA damage may cause PARP over-activation, leading to depletion of the cellular NAD^+^/ATP stores and occurrence of cell necrosis [9,10]. On the other hand, PARP activation induces PAR polymer formation primarily in the nucleus. PAR polymers can translocate to the mitochondria and mediate the release of AIF from the mitochondria [11-13]. Recent studies demonstrated that PARP-mediated AIF release is sequentially linked to calpain dependent AIF release [14,15]. AIF then translocate to the nucleus and induce cell apoptosis [7,16].

This study evaluates the effects of IH-induced oxidative stress on cell death, as well as the cell death pathways involved in these processes, in primary rat cerebellar granule cell cultures. Understanding the molecular mechanism involved in the regulation of IH-induced cell death will lead to better therapies for OSA-related syndromes.

## Methods

### Chemicals and solutions

Basal Medium Eagle, Fetal calf serum, and gentamycin were purchased from Gibco. DPQ was purchased from AXXORA. All fluorescent indicators were purchased from Molecular Probes (Eugene, OR). The TUNEL kit was purchased from Roche Molecular Biochemicals. All other chemicals were purchased from Sigma.

### Primary culture of cerebellar granule cells

All procedures were performed following the Animal Care Guidelines of Tzu Chi University. 7-day-old Sprague-Dawley rats (of either sex) were killed by cervical dislocation and then decapitated. The cerebella were removed, minced, and dissociated with 0.025% trypsin for 15 min at 37°C. The dissociated cells were suspended in basal modified Eagle's medium containing 10% fetal calf serum, 25 mM KCl, 2 mM glutamine, and 50 μg/ml of gentamycin, then plated on poly-L-lysine-coated, 12 mm coverslips and maintained in a humidified 5% CO_2 _incubator. Cytosine arabinoside (10 μM) was added 24 h after plating to kill and arrest the replication of non-neuronal cells, especially astrocytes. After 6-7 days of culture, the purity of the granule cells was generally greater than 90%.

### IH exposures

Cerebellar granule cells were placed in Plexiglas box chambers (FIRSTEK I-80, length 20 cm, width 20 cm, height 8 cm) and exposed to normoxia (**RA**; 20% O_2_, 5% CO_2_, and balance N_2_) or intermittent hypoxia (**IH**; 5% O_2_, 5% CO_2_, and balance N_2 _for 30 min alternating with 30-min RA) using a timed solenoid valve controlled by Fotek SC-260 for 1- 4 days. Oxygen levels in the chamber were continuously monitored by an oxygen detector.

### Cellular ROS assay

ROS were detected using 5-(and-6)-chloromethyl- 2',7'- dichlorodihydrofluorescein diacetate acetyl ester (DCFDA) and dihydroethidium (HE). Cells were loaded with 5 μM DCFDA for 30 min and with 10 μM HE for 15 min at room temperature.

### Assessment of cell death

Necrotic cell death analysis: cells were subjected to IH or RA for indicated time and then stained with 1.5 μg/ml PI for 20 min. After PI staining, cells were washed with NT three times then fixed with 10% formaldehyde for 1 h. Nuclei were labeled with 1 mg/ml Hoechst 33342 for counterstained.

Apoptotic cell death analysis: cells were subjected to IH or RA for indicated time and then examined for apoptosis with TUNEL assay (In Situ Cell Death Detection kit, Roche).

Granule cells were stained for 1 h at 37°C and identified using the anti-Tau monoclonal antibody and secondary antimouse IgG FITC conjugate (green fluorescence) or IgG TRITC conjugate (red fluorescence).

### Investigation of cell death pathways

#### Inhibitors of cell death pathways

Phenanthroline (Phe; 1 μM), 3-aminobenzamide (3-AB; 1 mM), and DPQ (10 μM), were pre-treat and follow by IH for experiments in the IH3day and IH4day groups. Then cell death was assessed.

#### Caspase-3

Cells were incubated with FITC-DEVD-FMK (1:300) at 37°C and then fixed in 10% formaldehyde. Nuclei were labeled using Hoechst 33342.

#### AIF

Cells were fixed in 10% formaldehyde for 1 h. Nuclei were labeled using Hoechst 33342. Cells were incubated with the anti-VDAC monoclonal antibody (1:500), anti-AIF monoclonal antibody (1:500) and secondary antimouse IgG FITC or TRITC (1:500) for 1 h at 37°C.

### Western blotting

Cells were lysed on ice with 200 μl of lysis buffer (50 mM Tris-HCl, pH 7.5, 0.5 M NaCl, 5 mM MgCl2, 0.5% Nonidet P-40, 1 mM phenylmethylsulfonyl fluoride, 1 μg/ml pepstatin, and 50 μg/ml leupeptin) and centrifuged at 10600 × *g *at 4°C for 10 min. Mitochondria were isolated using a mitochondria isolation kit for cultured cells, number 89874 (PIERCE). The protein concentrations in the supernatants were quantified using a BSA Protein Assay Kit. Electrophoresis was performed on a NuPAGE Bis-Tris Electrophoresis System using 30 μg of reduced protein extract per lane. Resolved proteins were then transferred to PVDF membranes. Membranes were blocked with 5% non-fat milk for 1 h at room temperature and then probed with the appropriate dilution of primary antibodies at 4°C overnight: β-actin, tau, VDAC and cytochrome *c *(chemicon). Activated caspase-3, PARP and AIF (cell signaling). Calpain1 (GeneTex). After the PVDF membrane was washed three times with TBS/0.2% Tween 20 at room temperature, it was incubated with the appropriate secondary antibody (goat anti-mouse or anti-rabbit, 1:10000) and labeled with horseradish peroxidase for 1 h at room temperature. All proteins were detected using Western Lightning™ Chemiluminescence Reagent Plus (Amersham Biosciences, Arlington Heights, IL).

### Confocal microscopy

Cells were observed using a laser scanning confocal microscope (TCS-SP, Leica). Images were analyzed using Leica confocal software.

### Statistics

The results of fluorescence measurements and cell proliferation experiments are expressed as the mean ± SEM. The *t*-test and one-way ANOVA with post-hoc test were performed to test differences between groups using the SPSS 18.0 software (SPSS Taiwan Corp.). All tests were considered to be statistically significant when P < 0.05.

## Results

### IH induced oxidative stress in rat primary cerebellar granule cells

**Figure 1:**
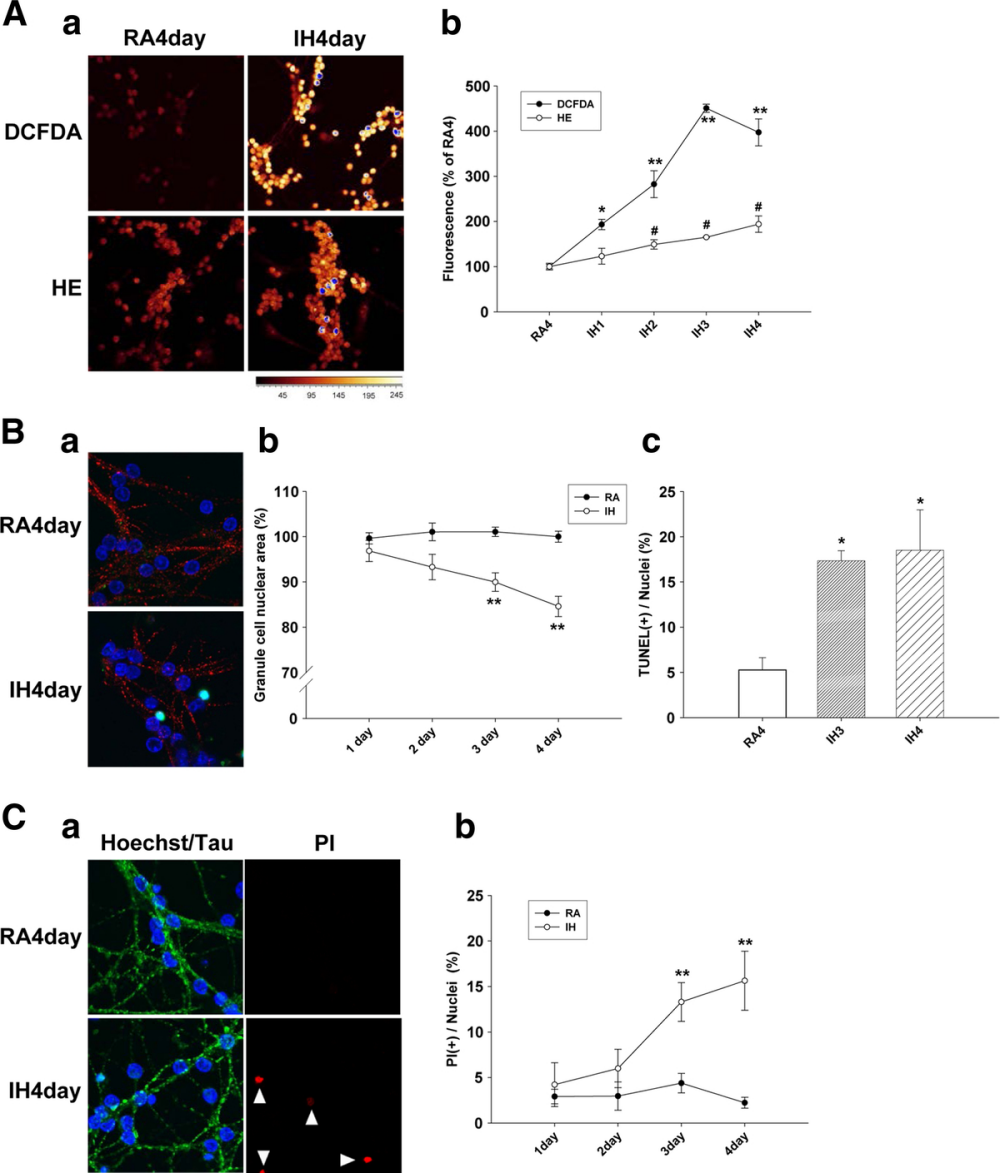
**IH increases ROS generation and induces cell death**. Aa, b: Images (shown with pseudocolor) and analysis of DCFDA and HE fluorescence staining for ROS. The average fluorescence of the RA4day group was set at 100%. *p = 0.058 and **p < 0.01 for comparing each group with RA4day group by Dunnett's test in ANOVA for DECFA. #p < 0.01 for comparing each group with RA4day group by Dunnett's test in ANOVA for HE. **Ba**: Cells were costained with Hoechst (blue), TUNEL (green), and Tau (red). Tau was as used as a neuronal marker. **Bb**: Quantitative assessment of apoptosis based on the area of the nuclei of cerebellar granule cells. **p < 0.01 for comparing each group with RA4day group by Dunnett's test in ANOVA for RA4day and IH1-4 day groups. **Bc**: Quantitative assessment of apoptosis based on the ratio of TUNEL (+) cells. *p < 0.05 for comparing RA3day with IH3day groups and IH4day by *t*-test. **Ca**: Cells were costained with Hoechst (blue), PI (red), and Tau (green). **Cb**: Quantitative assessment of apoptosis based on the ratio of PI (+) cells. RA4: RA4day group; IH3: IH3day group; IH4: IH4day group. **p < 0.01 for comparing each group with RA4day group by Dunnett's test in ANOVA for RA4day and IH1day-IH4day groups.

Oxidative stress progressively increased with increase in the duration of IH (Figure 1Aa, b). The average fluorescence of the RA (normoxia) 4 day group was set at 100%. In cells stained with HE (to detect O_2_^-^·), the average fluorescence of the IH1day-IH4day groups was 123.04 ± 17.64%, 149.11 ± 10.22%, 165.04 ± 1.0%, and 194.01 ± 18.12%, respectively. The fluorescence of the IH4day group was about twice that of the RA4day group. In cells stained with DCFDA (to detect H_2_O_2 _and OH·), the average fluorescence of the IH1day-IH4day groups was 193.39 ± 11.37%, 282.52 ± 29.69%, 450.76 ± 9.04%, and 397.27 ± 29.65%, respectively. The fluorescence of the IH3day and IH4day groups was about four times that of the RA4day group (Figure 1Aa, b). The fluorescence of the IH3day group and IH4day group was not significantly different (P = 0.106, LSD test in ANOVA).

### IH-induced apoptosis in rat primary cerebellar granule cells

Cell nuclei were visualized by Hoechst dye staining. Increase in the number of apoptotic cells resulted in decrease in the average area of the nuclei. The average area of the nuclei of the RA4day group was set at 100%. Compared to the RA4day group, the RA1day-RA3day groups was 99.63 ± 1.24%, 101.05 ± 1.97%, and 101.07 ± 1.03%, respectively. The IH1day to IH4day groups was 96.85 ± 2.34%, 93.28 ± 2.80%, 89.97 ± 2.04%, and 84.61 ± 2.23%, respectively. The average area of the nuclei among the RA1day-RA4day groups were not significantly different (P = 0.899, ANOVA); however, there were significant difference in the average area of the nuclei between the RA4day from IH2day, IH3day and IH4day groups (Figure 1Bb).

Colocalized TUNEL (green)- and Hoechst nuclear (blue)-stained areas indicate apoptotic cells (Figure 1Ba). The TUNEL (+) ratio in the RA3day and RA4day groups was 4.38 ± 1.59% and 5.27 ± 1.36%, respectively. The TUNEL (+) ratio in the IH3day and IH4day groups was 17.34 ± 1.12% and 18.51 ± 4.46%, respectively. There was a significantly higher TUNEL (+) ratio in the IH3day and IH4day groups than in the RA3day and RA4day groups, respectively (Figure 1Bc).

The IH groups exhibited higher levels of apoptosis, and this trend increased with increased exposure to IH.

### IH-induced necrosis in rat primary cerebellar granule cells

Colocalized PI (red)- and Hoechst nuclear (blue)-stained areas indicate necrotic cells and loss of membrane integrity (Figure 1Ca). The PI (+) ratio of the RA1day-RA4day groups was 2.92 ± 0.8%, 2.97 ± 1.55%, 4.39 ± 1.07%, and 2.23 ± 0.6%, respectively. The PI (+) ratio of the IH1day-IH4day groups was 4.22 ± 2.40%, 6.0 ± 2.11%, 13.31 ± 2.12%, and 15.64 ± 3.24%, respectively. The PI (+) ratios among the RA1day-RA4day groups was not significantly different (P = 0.899, ANOVA); however, there were significant differences in the ratios between the RA4day from IH3day and IH4day groups (Figure 1Cb). More necrotic cells were found in the IH groups, and this trend increased with longer durations of IH.

### IH-induced cell death can be rescue by inhibitors pretreatment

**Figure 2:**
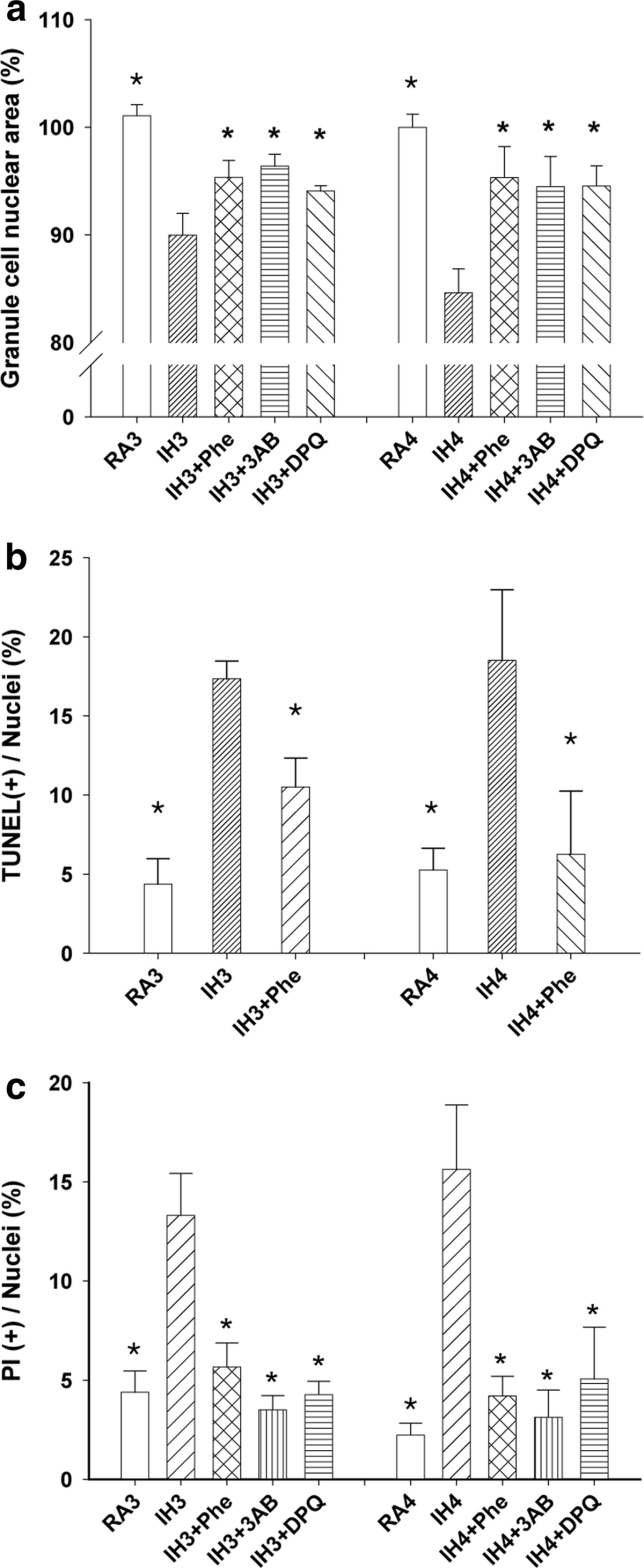
**IH-induced cell death was associated with oxidative stress and PARP activation**. A: Severe apoptosis appeared in the IH3day and IH4day groups. The administration of inhibitors significantly decreased the occurrence of apoptosis. **B**: There were more TUNEL (+) cells in the IH3day and IH4day groups. Addition of Phe resulted in a decrease in the ratio of TUNEL (+) cells. **C**: There were more PI (+) cells in the IH3day and IH4day groups. The ratio of PI (+) cells decreased significantly after the administration of inhibitors. *p < 0.05 for comparing each group with IH3day group or with IH4day group by Dunnett's test in ANOVA.

The average area of the nuclei of cells in the IH3day group was 89.97 ± 2.04%, and that in the IH3day group treated with Phe, 3-AB, and DPQ was 95.34 ± 1.58%, 96.38 ± 1.03%, and 93.44 ± 0.50%, respectively. The average area of the nuclei of cells in the IH4day group was 84.61 ± 2.23%, while that of cells in the IH4day group treated with Phe, 3-AB, and DPQ was 95.33 ± 2.88%, 94.48 ± 2.44%, and 91.90 ± 3.11%, respectively. The average area of the nuclei of cells increased significantly in the IH3day and IH4day groups treated with inhibitors (Figure 2A). The TUNEL (+) ratio of the IH3day and IH4day groups was 17.34 ± 1.12% and 18.51 ± 4.46%, respectively. Furthermore, the ratio of cells treated with Phe in the IH3day and IH4day groups was 10.50 ± 1.84% and 6.26 ± 3.98%, respectively. The ratio of both groups treated with Phe was significantly lower than that of the untreated IH3day and IH4day groups (Figure 2B). Apoptotic cell death decreased in the presence of inhibitors.

The PI (+) ratio of the IH3day group was 13.31 ± 2.12%, while that of the IH3day group treated with Phe, 3-AB, and DPQ was 5.66 ± 1.22%, 3.51 ± 0.71%, and 4.27 ± 0.67%, respectively. The PI (+) ratio of the IH4day group was 15.64 ± 3.24%, while that of the IH4day group treated with Phe, 3-AB, and DPQ was 4.21 ± 0.98%, 3.13 ± 1.38%, and 5.06 ± 2.62%, respectively. The ratio in the IH3day and IH4day groups treated with inhibitors decreased significantly (Figure 2C). Necrotic cell death decreased in the presence of inhibitors. These data suggested that reduction in oxidative stress or PARP inhibition resulted in the decrease in apoptosis and necrosis.

### Caspase-3 activation was not involved in IH-induced cell death

**Figure 3:**
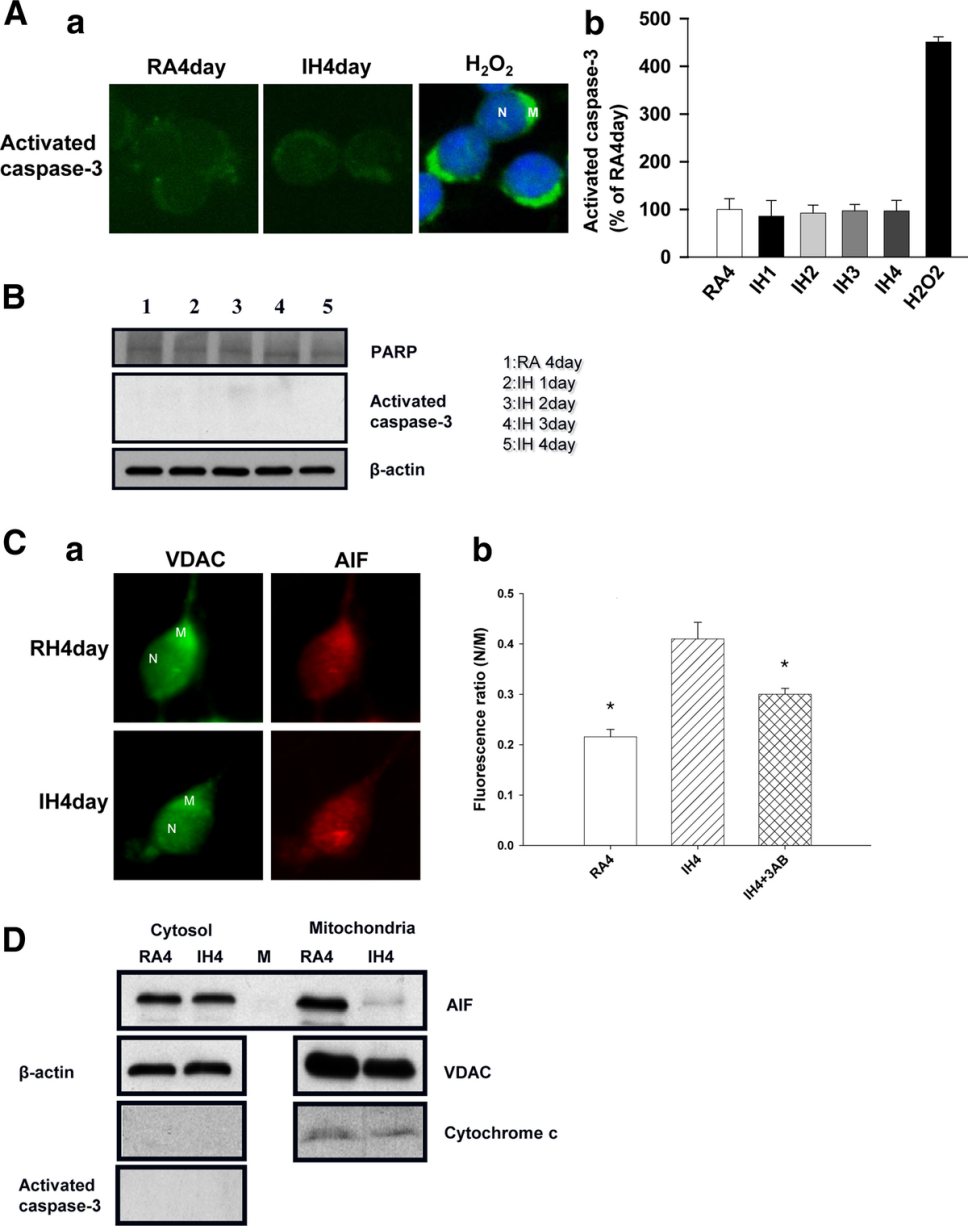
**IH-induced apoptosis is mediated by AIF translocation instead of caspase-3 activation**. Aa, b: Pictures and quantitative assessment of staining of the caspase-3-cleaved substrate FITC-DEVD-FMK. There was no significant difference between the RA and IH groups. **B**: Western blotting analysis of whole cell proteins showed no activated caspase-3 or cleaved PARP. **Ca**: Cells were costained with AIF and VDAC. N: nucleus; M: mitochondria. **Cb**: Quantitative assessment of AIF staining based on the ratio of N/M fluorescence. **D**: Western blotting analysis of subcellular fractions. The mitochondria did not release cytochrome c, and activated caspase-3 was not noted. AIF was released from the mitochondria in the IH4day group. β-actin was used as an internal control. VDAC was used as a loading control of mitochondrial fraction. M: marker. *p < 0.05 for comparing each group with IH4day group by Dunnett's test in ANOVA.

Caspase-3 cleaved the substrate FITC-DEVD-FMK, resulting in its fluorescence. The fluorescence of the RA4day group was set at 100%. The fluorescence of the IH1day-IH4day groups was 85.69 ± 32.78%, 92.24 ± 16.57%, 96.87 ± 13.30%, and 96.70 ± 22.12%, respectively. There were no significant differences in fluorescence among the RA4day and IH1day-IH4day groups. Cells treated with H_2_O_2 _served as the positive control, and their fluorescence was 451 ± 11.0%. (Figure 3Aa, b). Whole cell proteins of the RA4day and IH1day-IH4day groups were extracted for Western blotting analysis, with β-actin as the internal control. Results did not indicate the activation of caspase-3 or fragmentation of PARP, which was cleaved by caspase-3 in the caspase-dependent apoptotic pathway (Figure 3B). Therefore, IH did not induce caspase-3 activation.

### IH-induced cell death was correlated with AIF nuclear-translocation

Cells were co-stained with AIF and VDAC, a mitochondrial marker. The nucleus and mitochondria were labeled as N and M, respectively (Figure 3Ca). The ratio of fluorescence of the nucleus/mitochondria (N/M) was measured by AIF immunostaining. Increase in the N/M ratio was indicative of increased AIF translocation to the nucleus. The N/M ratio of the RA4day group was 0.21 ± 0.0065%, while that of the IH4day group was 0.41 ± 0.0109%. The N/M ratio of the IH4day group was significantly higher than that of the RA4day group. Inclusion of the PARP inhibitor 3-AB in the IH4day group decreased the N/M ratio to 0.30 ± 0.0047%, which was significantly different from the ratio of the untreated IH4day group (Figure 3Cb). Cells of the RA4day and IH4day groups were subjected to subcellular fractionation, and immunoblotting was performed on the cytosolic and mitochondrial fractions (Figure 3D). β-actin and VDAC were used as cytosolic and mitochondrial loading control, respectively. The amount of AIF in the mitochondria of the cells in the IH4day group seemed to be less than that in the RA4day group, indicating that AIF was released from the mitochondria and translocated to the nucleus. The amount of cytochrome *c *expressed in the mitochondria of the cells in the RA4day and IH4day groups was the same. Cytochrome *c *and activated caspase-3 were not detected in the cytosol. Similar to the above observation (Figure 3A, B), since cytochrome *c *was not released from the mitochondria into the cytosol, it did not induce the activation of caspase-3.

### PARP inhibition abrogates calpain's activation

**Figure 4:**
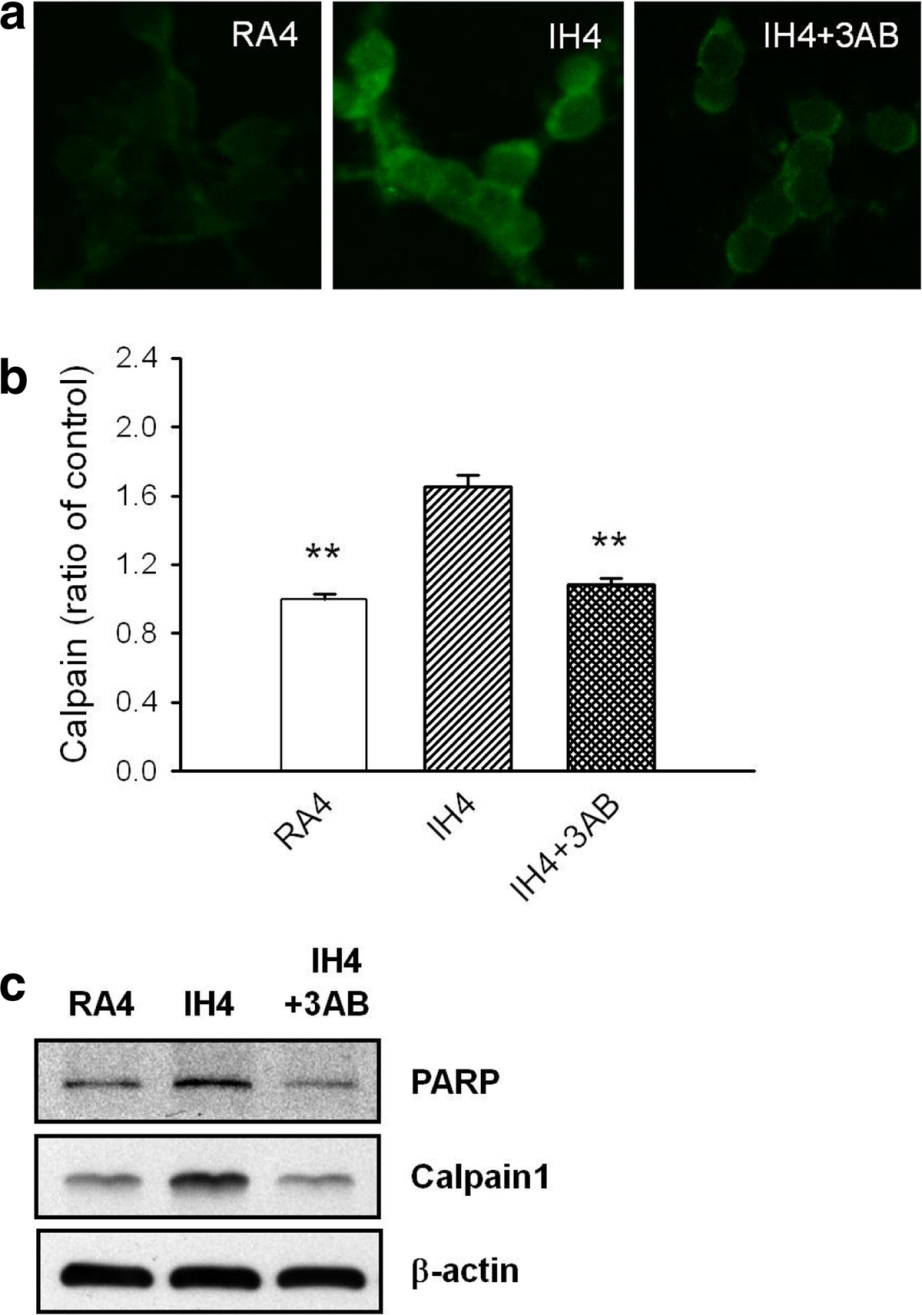
**IH-induced cell death was associated PARP-mediated calpain activation**. **A**: Immunostaining for calpain1 in RA4, IH4 and IH4 treated with 3AB groups. **B**: Quantitative assessment of immunostaining data from figure 4A. **C**: Western blot analysis of PARP and calpain was performed in the RA4, IH4 and IH4 treated with 3AB groups. β-actin was used as an internal control. **p < 0.01 for comparing each group with IH4day group by Dunnett's test in ANOVA.

Exposure cells to IH4 day resulted in elevated calpain expression which was blocked by using PARP inhibitor 3-AB (Figure 4A). The quantitative data of Figure 4A showed that calpain-positive ratio in the IH4day group was 1.65 ± 0.063% fold higher than RA4day group, and decreased to 1.08 ± 0.03% fold in the IH4day treated with 3-AB group (Figure 4B). We validated the up-regulation of calpain by western blot. IH elicited an increased expression of calpain proteins that was diminished in IH4day treated with 3-AB group (Figure 4C).

## Discussion

Intermittent hypoxia [1] has been shown to increase oxidative stress and/or reduce anti-oxidative capacity [17,18]. This study shows that ROS accumulation in cells is proportional to the duration of IH. IH is different from sustained hypoxia; however, it has the analogous process of hypoxia-reoxygenation as ischemia-reperfusion. Large amounts of ROS are generated during the transition from hypoxia to normoxia. Excessive ROS interact with nucleic acids, lipids, and proteins, resulting in cellular damage and death.

Our results demonstrate that IH causes oxidative stress and induces cell death in rat cerebellar granule cells. IH-induced cell death can be partially rescue by Phenanthroline pre-treatment. Xu et al. reported that chronic IH causes oxidative stress and increases apoptosis in the cortical neurons of mice. However, apoptosis decreased when transgenic mice overexpressing Cu/Zn superoxide dismutase were exposed to the same conditions [19]. These data indicate that oxidative stress induced by IH is related to IH-induced cell death.

We further investigated the roles of necrosis in IH-induced cell death in granule cells. Neither NAD^+ ^nor ATP was detected in these cells, and cell death was partially rescued by PARP inhibitors (3-AB and DPQ) pre-treatment. Taken together, IH-induced necrotic cell death is associated with PARP activation.

Gozal et al. reported that IH induced more severe apoptosis than sustained hypoxia via the caspase-3-dependent pathway in PC-12 cells [20]. In this study, neither activation of caspase-3 nor the cleavage of PARP protein was observed after IH-induced cell death. These results reveal that IH induces apoptotic cell death through caspase-3 independent pathway.

Both apoptotic and necrotic cell death induced by IH were decrease in the presence of PARP inhibitors. Therefore, apoptotic cell death was believed to be associated with PARP activation. The amount of AIF translocation to the nucleus increased in the IH4day group, and decreased in the presence of PARP inhibitor 3-AB, while cell death were also decreased after the 3-AB pre-treatment. In summary, the mechanism of IH-induced apoptosis in cerebellar granule cells was regulated by PARP-mediate AIF activation and was caspase-3- independent.

In previous reports, caspases were activated by IH-induced apoptosis [20], but caspase activation was not noted in our study. Owing to most studies on IH-induced apoptosis using PC-12 cells or other neural cells instead of cerebellar granule cells, this finding may be correlated with using different cell types. Recently, some reports indicated that the mechanism of IH-induced apoptosis was different between cerebellar granule cells and other cells. Fonfria et al. [21] stated that AIF translocation to the nucleus results in the apoptosis of cerebellar granule cells exposed to neurotoxic agents such as H_2_O_2_. Liu et al. [22] demonstrated that c-Jun N-terminal kinase (JNK) is involved in hypoxia and reoxygenation-induced apoptosis of cultured rat cerebellar granule neurons. PARP-mediated cell death is associated with activation of JNK, which contributes to mitochondrial dysfunction [23], and translocation of AIF. In addition, Vosler et al. demonstrated that activation of PARP-1 is necessary for calpain activation as PARP-1 inhibition bloacked mitochondrial calpain activation [24]. They suggested that PARP-1 and calpain act in concert following calcium dysregulation to induce AIF release during ischemia [14,15]. Taken together, our data suggested that IH-induced PARP activation flowed by calpain activation and subsequent AIF-mediated caspase-independent apoptosis in rat cerebellar granule cells.

Yang et al. reported that oxidative stress induces apoptosis and necrosis in a single cultured rat cardiomyocyte [25]. The present study shows that oxidative stress by IH induces both modes of cell death in primary cerebellar granule cells. High levels of ROS result in over-activation of PARP in the nucleus. NAD^+ ^and ATP depletion results in cell necrosis. PAR polymers mediate AIF release and AIF-induced apoptosis. IH-induced ROS accumulation resulted in increasing cell damage. Severe cell damage depletes NAD^+ ^and leads to the production of more PAR polymers, ultimately resulting in increased cell necrosis and apoptosis. Therefore, it is believed that over-activation of PARP resulting from accumulated ROS leads to both cell necrosis and apoptosis.

## Conclusions

**Figure 5:**
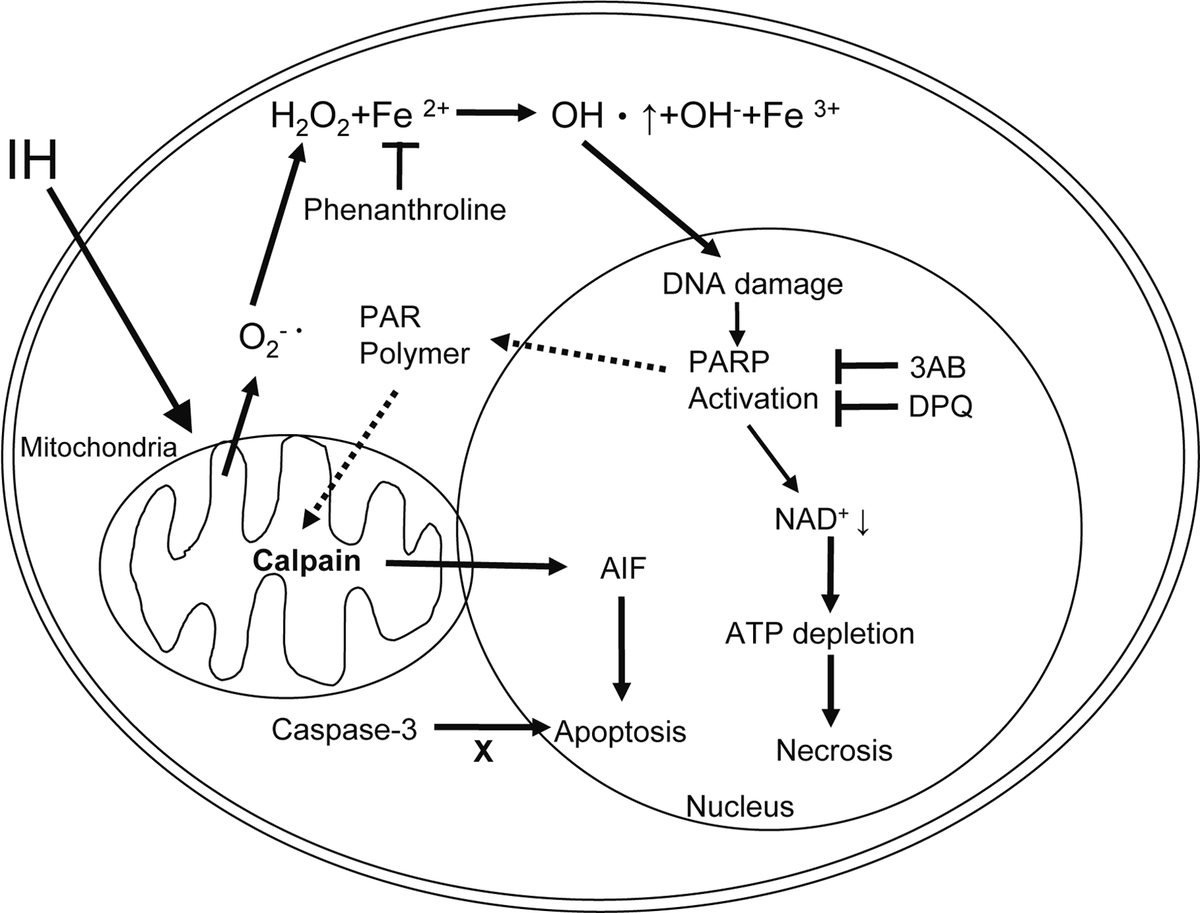
**Possible molecular mechanism for IH-induced cell death in rat cerebellar granule cells**. IH causes elevated oxidative stress and induces cell apoptosis and necrosis. Overactivation of PARP causes ATP depletion, leading to cell necrosis. PARP activation also generates PAR polymers, activates mitochondrial calpain, which induce AIF translocation to the nucleus and lead to cell apoptosis.

Our studies point out the roles of PARP activation in IH-induce oxidative stress and cell death in cerebellar granule cells. Over-activation of PARP causes ATP depletion, calpain activation and AIF translocation, thus leading to apoptosis and necrosis (Figure 5).

## Abbreviations

IH: intermittent hypoxia; PARP: poly (ADP-ribose) polymerase; AIF: apoptosis-inducing factor.

## Competing interests

The authors declare that they have no competing interests.

## Authors' contributions

Conceived and designed the experiments: SYH, SCC, KTY. Performed the experiment: SYH, SCC, YCT, CFL, YJL. Contributed reagents/materials/analysis tools: SCC, KTY. Analyzed the data: SYH, SCC, SPC, YCT. Wrote the paper: SYH, SCC, CYP, KTY. All authors read and approved the final manuscript.
